# Effect of the Thermal History on the Crystallinity of Poly (L-lactic Acid) During the Micromolding Process

**DOI:** 10.3390/mi11050452

**Published:** 2020-04-25

**Authors:** Hiroaki Takehara, Yuki Hadano, Yukihiro Kanda, Takanori Ichiki

**Affiliations:** 1 Department of Materials Engineering, School of Engineering, The University of Tokyo, 7-3-1 Hongo, Bunkyo-ku, Tokyo 113-8656, Japan; hadano@bionano.t.u-tokyo.ac.jp (Y.H.); kanda@bionano.t.u-tokyo.ac.jp (Y.K.); ichiki@bionano.t.u-tokyo.ac.jp (T.I.); 2 Innovation Center of NanoMedicine (iCONM), 3-25-14 Tonomachi, Kawasaki, Kanagawa 210-0821, Japan

**Keywords:** semi-crystalline polymer, PLLA, thermoplastics, microdevice

## Abstract

The micromolding process using biocompatible thermoplastic polymers is highly attractive as a fabrication process of microdevices in biomedical applications. In this study, we investigated the effect of the thermal history in the micromolding process on the crystallinity of semi-crystalline polymers, such as poly (L-lactic acid) (PLLA), during their crystallization from the amorphous and molten states. In particular, the thermal history in the micromolding process using poly(dimethylsiloxane) replica mold embedded with a thermocouple was recorded. The crystallinity of PLLA constructs fabricated using the micromolding process was measured via wide-angle X-ray scattering, and crystallization kinetics was analyzed based on the Kolmogorov–Johnson–Mehl–Avrami equation. A crystallization rate of *k* = 0.061 min^−*n*^ was obtained in the micromolding process of PLLA crystallization from the amorphous state, accompanied by the quenching operation, forming a large number of crystal nuclei. Finally, the fabrication of PLLA microneedles was performed using micromolding processes with different thermal histories. The information about the thermal history during the micromolding process is significant in the development of polymer microdevices to achieve better material properties.

## 1. Introduction

Bioabsorbable polymers are useful as materials for constructing medical devices used in the human body. Furthermore, the development of precision processing and microfabrication techniques for device miniaturization is in progress. Poly (lactic acid) polymers such as PLLA are approved by the US Food and Drug Administration (FDA) as generally recognized as safe (GRAS) [1]. Therefore, poly (L-lactic acid) (PLLA) is one of the most promising thermoplastic materials for biomedical applications, including surgical sutures, implants, and microneedles [2,3].

The micromolding process is an established manufacturing technology for fabricating biomedical microdevices using thermoplastic polymers. The production cost of the micromolding process mainly relies on the fabrication cost for the master mold, and thus, the cost of the employed material is negligibly low. This aspect of the micromolding process enables the use of high-end materials (e.g., the pharmaceutical grade) even for disposable usage [4]. However, polymer materials change their material properties depending on the process conditions, especially the thermal history during the molding process [5,6].

The information regarding the fabrication process and material properties can be used as a guideline for the development of polymer microdevices to ensure their excellent material properties. In particular, semi-crystalline polymers including PLLA change their crystallinity during the micromolding process. It has been found that the degree of crystallinity of PLLA impacts the hydrolytic degradation kinetics inside the human body in clinical applications [7]. The mechanism of the hydrolytic degradation could be considered as the combination of chemical hydrolysis of polymer chain scission at the ester bond [8] and the diffusion of water molecules and divided oligomers via the bulk-erosion mechanism [9]. The hydrolysis of PLLA predominantly occurs in the amorphous region [10] because of the difficulty of the diffusion of water molecules into the rigid crystalline region [11]. The degree of crystallinity also determines their physical properties, including mechanical behavior. The mechanism of the deformation of the amorphous and crystalline PLLA polymers indicates that the amorphous region could be deformed by crazing, and the crystalline region could show cavitation and fibrillated shear [12]. Crystalline PLLA materials often show a higher modulus of elasticity than amorphous PLLA materials [13]. In contrast, amorphous PLLA materials show higher bending strength than crystalline because of the plastic deformation after reaching their yield point [14].

Although previous research well-argued and disclosed the correlation between the crystallinities of PLLA and the material properties of hydrolysis and mechanical behavior, there is limited research about the implementation of the above knowledge into the micromolding process for fabricating biomedical microdevices. Harris and Lee reported that adding talc and ethylenebis-stearamide (EBS) increases the crystallization rate in the injection molding process [15]. This finding could indicate that the talc and EBS function as a physical nucleation agent, and thus, shortens the nucleation and crystal growth rate. Although such an approach is feasible to use as environmentally friendly materials, it might not be feasible in clinical use because of the necessity to guaranty the safety of the added materials. Iozzino et al. reported that crystalline regions show better resistance to hydrolysis than amorphous regions in the micromolded pure PLA biphasic samples [16]. However, the crystallization rate of the PLLA in the micromolding process and the difference in the thermal histories (i.e., crystallization processes from the amorphous state and the molten state) have not been investigated, despite the importance of this information for fabricating biomedical microdevices, such as microneedles. To obtain polymeric devices with sufficient crystallinity to control optimal physical and chemical properties, the information about the crystallization rate in the micromolding process has importance.

To understand how to control the crystallinity of polymer materials fabricated using the micromolding process, we investigated the effect of the thermal history on the crystallinity of PLLA during the micromolding process. The findings of this study can be applied to the fabrication process of microdevices for biomedical applications. The crystallization of semi-crystalline polymers during the micromolding process can be classified into two main types: (i) the crystallization from the molten state and (ii) the crystallization from the amorphous (glassy) state. This study investigates the effect of the thermal history during the two types of the crystallization process on the crystallinity and crystallization kinetics of PLLA.

## 2. Materials and Methods

### 2.1. Materials

PLLA pellet (Lot # STBH0071, Resomer L 206 S, Sigma-Aldrich Corp., St. Louis, MO, USA) and a prepolymer of poly(dimethylsiloxane) (PDMS) (Sylgard 184, Dow Corning Co., Midland, MI, USA) were used in this study. The PLLA pellet was characterized by gel-permeation chromatography (GPC) analysis. Monodisperse polystyrene standards (Sigma-Aldrich Corp., St. Louis, MO, USA) were used for calibration using GPC KF-804L column (Shodex, Showa Denko K.K., Tokyo, Japan) with tetrahydrofuran (THF, Fujifilm Wako Pure Chemical Corp., Tokyo, Japan) as an eluent at 40 °C and a flow rate of 1 mL/min. The molecular weight (M_w_) and polydispersity index (M_w_/M_n_) of the PLLA pellet were evaluated as M_w_ = 12.1 kg/mol and M_w_/M_n_ = 1.34, respectively. The PDMS prepolymer was prepared using the mixing ratio of BASE:CAT = 10:1.5. To form molds for the micromolding process, the prepolymer solution was cured at 85 °C for 2 h.

### 2.2. Sample Preparation with Different Thermal History in the Micromolding Process

**Figure 1:**
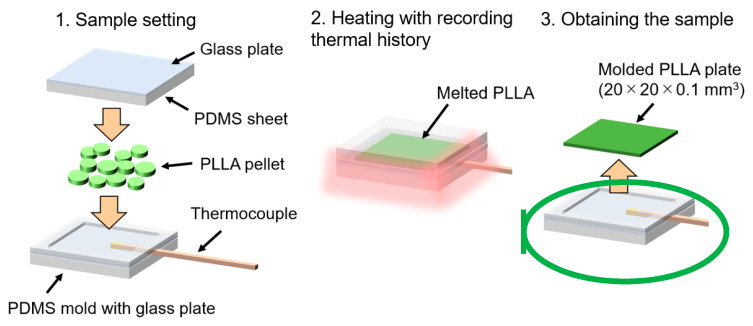
Schematic illustration of the poly (L-lactic acid) (PLLA) micromolding process with recording its thermal history using a poly(dimethylsiloxane) (PDMS) replica mold.

The change in the crystallinity of the PLLA due to the temperature change during molding was examined using a PLLA flat plate sample. Figure 1 shows the PLLA micromolding process, including its thermal history. The PLLA pellet was melted on a PDMS mold and molded into the PLLA flat plate sample (width 20 mm, height 20 mm, thickness 0.1 mm). The process temperature was measured using a thermocouple (TCTG022, Sakaguchi E.H VOC Corp., Tokyo, Japan) embedded inside the PDMS mold. To record the thermal history during the micromolding process, the thermocouple was connected to a thermometer (BAT-10, Physitemp Instruments LLC, Clifton, NJ, USA) and a multimeter (7461A, ADCMT Corp., Tokyo, Japan). 

Crystallization of semi-crystalline polymers can be classified into two processes of crystallization from the molten and amorphous states. Therefore, micromolding processes with two different thermal histories were performed (referred to as process (i) and process (ii)). In process (i), the mold was maintained at the crystallization temperature (120–130 °C) for 2.5, 5.0, 7.5, 10.0, 12.5, 15, and 20 min after PLLA melting and then cooled to room temperature. In process (ii), the mold was rapidly cooled after PLLA melting to 40–50 °C, which is below T_g_ of PLLA, and then heated to the crystallization temperature (120–130 °C) and held for the crystallization time *t* = 3.0, 4.0, 5.0, 7.5, 10, and 15 min. For preparing the samples with a crystallization time of 0 min, the mold was rapidly cooled (>200 °C/min) to room temperature (20–25 °C) after PLLA melting. 

### 2.3. Microneedle Fabrication Using the Micromolding Process

**Figure 2:**
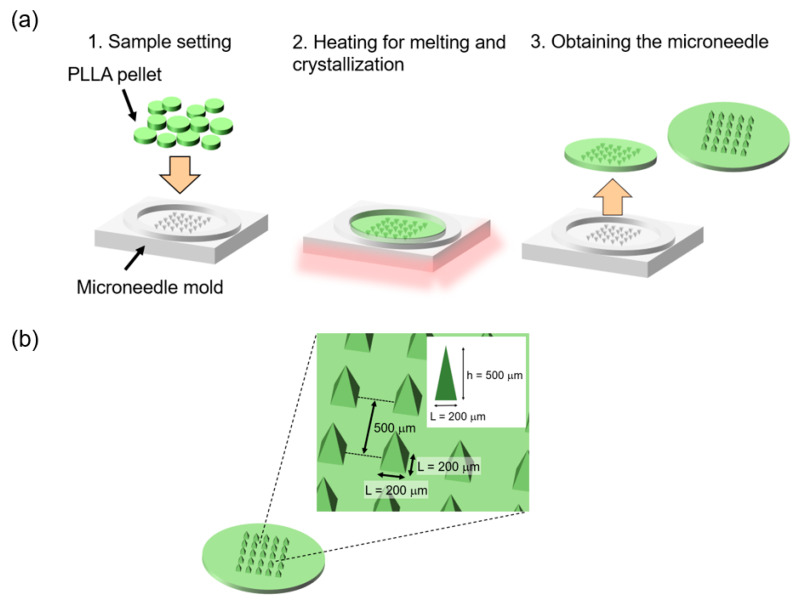
(**a**) Schematic illustration of the procedures of the micromolding process for fabricating the PLLA microneedles. (**b**) The dimensions of the fabricated PLLA microneedles.

Figure 2a shows the fabrication process of microneedles using the micromolding process. In the fabrication process of the microneedles with a crystallization time of 0 min, the PLLA pellet was melted into the mold at 220–240 °C, and then, rapidly cooled (>200 °C/min) to room temperature (20–25 °C). In the fabrication process of the PLLA microneedles with crystallization from the molten state (crystallization time *t* = 20 min), the PLLA pellet was melted into the mold at 220–240 °C, and then, the mold was maintained at the crystallization temperature (120–130 °C) for 20 min. Finally, the mold was cooled to room temperature (20–25 °C). A microneedle mold (ST-17, Micropoint Technologies Pte Ltd., Singapore) was used for the fabrication of microneedles with different thermal histories. Figure 2b indicates the microneedle mold dimensions used in the micromolding process. 

### 2.4. Characterization

Wide-angle X-ray scattering (WAXS) analysis was performed on the PLLA flat samples using Cu-Kα X-ray sources (λ = 0.154 nm), working at 40 kV and 30 mA, a step size of 0.05 deg, and a scan speed of 20 deg/min (SmartLab, Rigaku Corp., Tokyo, Japan). WAXS analysis was also performed on the PLLA microneedles using the Cu-Kα X-ray sources working at 40 kV and 15 mA, a step size of 0.02 deg, and a scan speed of 10 deg/min (MiniFlex600, Rigaku Corp.). The crystallinity (%) was estimated from *I_c_*/(*I_c_* + *I_a_*), where *I_c_* denotes the diffraction intensity derived from crystalline state and *I_a_* denotes the diffraction intensity derived from the amorphous state from the WAXS spectra, using a software (PDXL, Rigaku Corp.) [17]. 

## 3. Results

### 3.1. Thermal History

**Figure 3:**
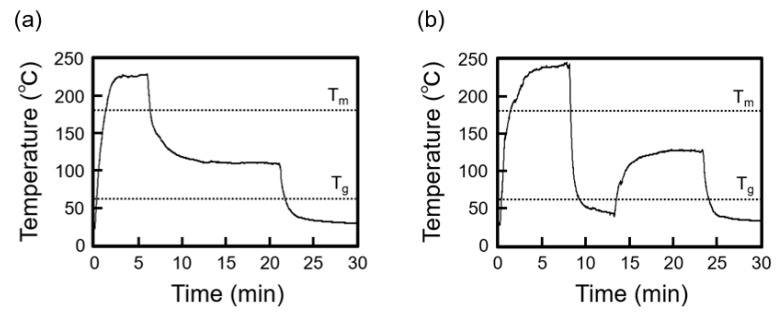
Temperature during the micromolding process. (**a**) After PLLA melting, it was maintained at crystallization temperature (120–130 °C) and then cooled to room temperature (micromolding process (i)). (**b**) After PLLA melting, it was rapidly cooled from >200 °C/min to below T_g_ and then heated to crystallization temperature (micromolding process (ii)).

The PLLA plate samples were formed using the micromolding process (i) with crystallization from the molten state and (ii) with crystallization from the amorphous state. Figure 3a,b shows the representative thermal histories obtained with processes (i) and (ii), respectively. In process (i), the temperature of the PDMS mold was kept over T_m_ to form the molten state of PLLA, and then the PDMS mold was kept at crystallization temperature (120–130 °C) and cooled to room temperature. In process (ii), the temperature of the PDMS mold was kept over T_m_ and then rapidly cooled below T_g_ to form the amorphous state of PLLA. The rapid cooling rate of >200 °C/min was measured to form the amorphous state in process (ii) as shown in Figure 3b. 

### 3.2. Crystallinity

**Figure 4:**
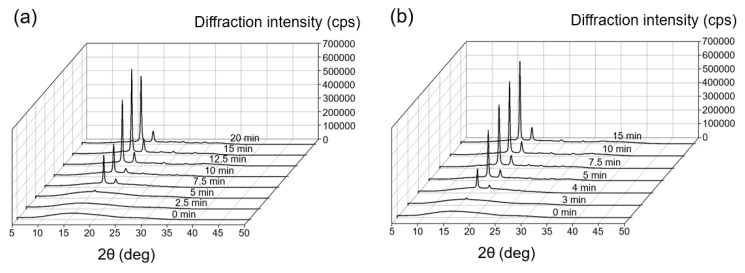
X-ray diffraction peaks obtained from the tested micromolded PLLA samples. (**a**) PLLA formed in micromolding process (i) with crystallization from the molten state. (**b**) PLLA formed in micromolding process (ii) with crystallization from the amorphous state.

Figure 4a shows the WAXS pattern of PLLA for the thermal history of process (i). The crystallization times were 2.5, 5.0, 7.5, 10.0, 12.5, 15, and 20 min. Figure 4b shows the WAXS pattern of PLLA for the thermal history of process (ii). The crystallization times were 3.0, 4.0, 5.0, 7.5, 10, and 15 min. The WAXS pattern of PLLA without the crystallization time, which is characterized by a broad band with the maximum value at 2θ = 16.6°, indicates a complete amorphous state (Figure 4a,b, indicated as 0 min). The WAXS patterns of PLLA for the thermal histories of processes (i) and (ii) demonstrate sharp peaks at 16.7° and 19.1°, respectively (Figure 4a,b). The intensities of each peak increase depending on the increase of the crystallization time. The peaks at 16.7° and 19.1° were derived from the reflections of 110/200 and 203 planes of the orthorhombic unit cell of the α-form crystal structure of PLLA, respectively [18]. 

### 3.3. Crystallization Kinetics

**Figure 5:**
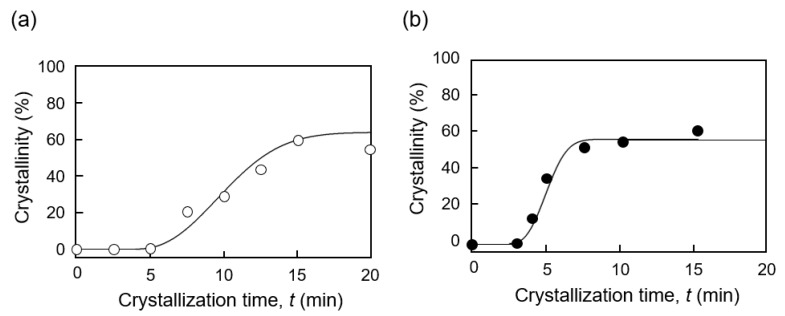
Crystallinity change with time determined using WAXS data. (**a**) PLLA formed in the micromolding process (i) with crystallization from the molten state. Chi-square value $X^{2}$ = 34.7. (**b**) PLLA formed in the micromolding process (ii) with crystallization from the amorphous state. Chi-square value $X^{2}$ = 20.2.

**Figure 6:**
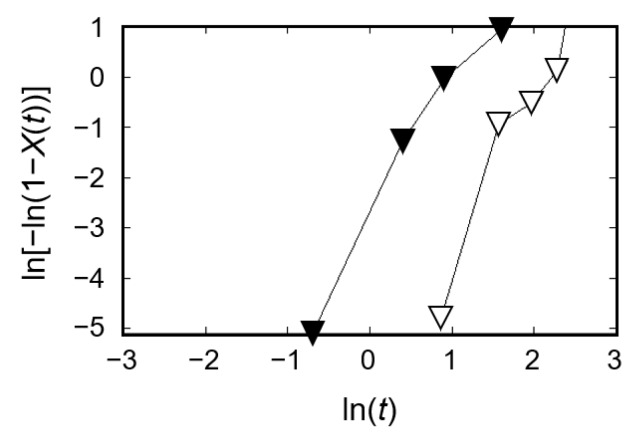
Avrami plots obtained from wide-angle X-ray scattering (WAXS) data: micromolding process (i) with crystallization from the molten state is represented with white triangles, while micromolding process (ii) with crystallization from the amorphous state is represented with black triangles.

Figure 5 shows the degree of the crystallinity (%) of the PLLA that was formed during micromolding processes (i) and (ii). The plots were fitted using the Kolmogorov–Johnson–Mehl–Avrami (KJMA) equation as [19,20]

(1) $$X\left(t\right)=X_{\infty }\left[1-\exp\left\{-k{\left(t-\tau \right)}^{n}\right\}\right],$$

where X(t) denotes the crystallinity at time *t*; $X_{\infty }$ denotes the crystallinity after infinite time; *k* denotes the overall crystallization rate constant depending on the nucleation and crystal growth rate; *n* denotes the Avrami exponent; and τ denotes the induction period considered as the period required to form a critical nucleus [21]. Figure 6 shows the plots of ln[−ln(1 − X(t))] vs. ln(*t*) for the micromolded PLLA substrates during processes (i) and (ii). It is widely known that the inhomogeneous distribution of nuclei results in nonlinearity of the plots, particularly at high-volume crystallinity X(t) [20]. Thus, the curves present a nonlinear end part; however, only the parts used to perform the fitting are shown in Figure 6 [21]. The induction time τ was considered to be 4.0 min for process (i) and 2.5 min for process (ii). The crystallization parameters *n* and *k* were 2.3 and 0.011 min^−*n*^ for process (i) and 2.7 and 0.061 min^−*n*^ for process (ii), respectively. The *n* and *k* values obtained during the micromolding process were consistent with those previously reported for the crystallization of PLLA obtained using differential scanning calorimetry (DSC) data [22,23,24]. The Avrami exponent value *n* in the range of 2–3 indicated that mainly a two-dimensional crystal growth was favored [25]. In contrast to process (i), an increase of the overall crystallization rate constant *k* was obtained during process (ii), accompanied with the quenching operation after PLLA melting. It is considered that the quenching operation formed a large number of crystal nuclei, and thus, the overall crystallization rate constant *k* increased [26]. 

### 3.4. Demonstration of Microneedle Fabrication with Different Thermal History

**Figure 7:**
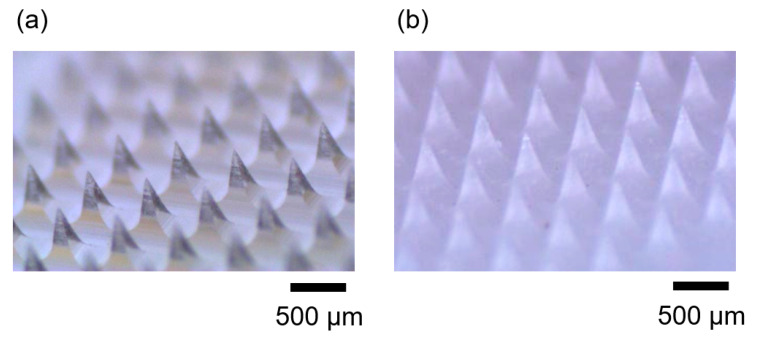
Photographs of the PLLA microneedle arrays fabricated using micromolding processes with different thermal histories. (**a**) PLLA microneedle array formed during micromolding with a rapid cooling process (>200 °C/min) from the molten state; crystallization time *t* = 0 min. The crystallinity was evaluated as 0.1%. (**b**) PLLA microneedle array formed in the micromolding process with crystallization from the molten state; crystallization time *t* = 20 min. The crystallinity was evaluated as 53.9%.

Finally, we demonstrated the fabrication of microneedles using the micromolding process with different thermal histories. Microneedles are widely used in biomedical microdevices for the drug delivery and vaccination technology [27,28,29,30,31,32,33,34]. The fabrication process of microneedle arrays was performed using the same procedure of forming PLLA sheet samples. PDMS replica molds were used to form microneedles with a needle height of 500 μm and a needle squared base of 200 μm. Figure 7 shows the PLLA microneedle arrays fabricated using the micromolding process with two different thermal histories. The microneedle fabricated with a crystallization time of 0 min was constructed using amorphous PLLA (Figure 7a). WAXS analysis evaluated the crystallinity 0.1%. The PLLA microneedle fabricated with crystallization from the molten state and a crystallization time of 20 min was mainly constructed using crystalline PLLA (Figure 7b). The crystallinity was evaluated as 53.9%. These structural differences of polymer materials due to the differences in the thermal histories of the micromolding process often affect material properties, such as the degradation rate and mechanical strengths. Therefore, the thermal history during the micromolding process should be considered to obtain biomedical microdevices with well-adapted material properties.

## 4. Discussion

Crystallization kinetics is critical when considering the productivity of fabricating products. A slow crystallization rate would result in long processing time in fabricating products. An extremely long processing time would be impractical and economically unfeasible for mass production. Therefore, increasing the crystallization rate in the micromolding process is critical. Controlling the crystallization rate is interesting and several techniques have been investigated by blending specific fillers [15,21]. Such approaches are effective and practical for use in the automotive, electronic, and agriculture sectors as environmentally friendly materials. However, they might be unfeasible in medical applications such as sutures, implants, and microneedles because the safety of the additive materials must be guaranteed. This study showed the crystallization rates of pure PLLA in two main types of crystallization processes during the micromolding fabrication technique, namely crystallization from the molten state, process (i), and crystallization from the amorphous state, process (ii). The crystallization process from the amorphous state (process (ii)), showed a high crystallization rate of 0.061 min^−*n*^ and might be favorable to shorten the crystallization process time to obtain the fully crystallized products. On the other hand, the crystallization process from the molten state (process (i)) might make it easy to control the crystallinity by controlling the crystallization time and fabricate products with a middle range of about 10%–40% crystallinity. 

## 5. Conclusion

This study investigated the effect of the thermal history during the micromolding process on the crystallinity of a semi-crystalline polymer, namely, PLLA. Two micromolding processes with crystallization from the amorphous and molten states were performed, and their thermal histories were recorded. The crystallinity of PLLA was obtained using WAXS, and crystallization kinetics was analyzed according to the KJMA equation. Compared to the micromolding process with crystallization from the molten state, a crystallization rate of *k* = 0.061 min^−n^ was obtained during the micromolding process with crystallization from the amorphous state, accompanied with the quenching operation forming a large number of crystal nuclei. Finally, PLLA microneedles were fabricated using the micromolding process with two different thermal histories. The thermal history during the micromolding process is important in the fabrication of polymeric microdevices with desired material properties because of its ability to change polymer material morphology.
